# Pneumonia and Malaria

**Published:** 1855-10

**Authors:** Floyd Morse

**Affiliations:** Bradford, Steuben Co., N. Y.


### ART. II.—*Pneumonia and Malaria.* By FLOYD MORSE, M. D., Bradford, Steuben Co., N. Y.

Theory is worth nothing, only so far as it teaches *practical facts.* This is an emphatic truth in medicine. It matters little if Dr. La Roche establishes the non-malarial origin of pneumonia, or not; only, that every practitioner is made acquainted with the important fact taught by Dr. Upshur—that "without the aid of quinine, they will lose one-half their cases of pneumonia, in malarial districts." This is as true of the malarial districts of New York as of Virginia and North Carolina. There can be few intelligent physicians of the present day, who have not noted the special influence of malaria on pneumonia, where it occurs in malarial regions. This is strictly true of those practitioners located in miasmatic districts. And he must be a dull observer, who does not see plain indications,—nay, *peremptory demands,* for treatment wholly inapplicable to pneumonia occurring in non-malarial districts. Yet, there are many physicians in western New York, enjoying a liberal practice, who invariably associate pneumonia with the lancet, calomel, and tartar emetic. This is the *important error.* Dr. Upshur's remark is the *important truth.* Physicians who have always practiced in upland regions, where the influence of marsh miasm is not known, cannot be made to appreciate the value of quinine in the treatment of the pneumonia of malarial regions, until they have attempted the management of such cases without it. Of this fact I have had experience. Accustomed for four years to the uncomplicated pneumonia of the upland districts of Livingston Co., I located two years since, in one of the valleys of Steuben Co., notorious for its "ague."

Feb. 22d, 1853, was called to see Miss H., aged 19 years. She had just recovered from a severe chill, and was then suffering from pain in the right side, fever, and cough, with bloody expectoration. Headache and dyspnœa were present. Physical exploration of the chest showed the inferior lobe of the right lung engorged, with some pleuritic tenderness. Prescribed calomel and ipecac, each ten grains, as an emeto-cathartic, and ordered vinegar fomentations to the chest. This at evening. Next morning found patient apparently much better: cough slight, expectoration free and less colored; dyspnœa less; pain in the side and headache diminished; thirst abated, and skin soft. Gave solution of emetic tartar, with mucilage of slippery elm.

In the afternoon was called to my patient in haste. Found her with intense fever; constant cough, and no expectoration; severe and constant pain in the side, with urgent dyspnœa. Placed my patient in the upright position, and took 12oz. blood from the arm, which induced syncope. Reäction established, I immersed the feet in warm water, took 2oz. of blood from the chest with cups, and continued the fomentations, with evaporating lotion to the head. At midnight the symptoms were abated; and morning found my patient with free expectoration, of a mucus character; respiration easier; pain and fever diminished. At 10 o'clock, A. M., the paroxysm returned with renewed vigor, and an aggravation of all the local symptoms. At 4 o'clock, P. M., another remission; but not bringing the same local relief the former remissions had afforded. At 10 o'clock, A. M., of the following morning, another paroxysm, of alarming severity, occurred, with great anxiety and intense local distress. Thus the case proceeded for five days: two remissions daily, each subsequent one less perfect and of shorter duration than the preceding,—alternating with paroxysms of increasing severity, each successive one aggravating the local difficulty and extending the area of inflammation, until I lost all confidence in treatment, and thought I must surrender my patient to the fury of the paroxysms. Delirium had now supervened: sawing the air with the hands; picking the bed clothes; bloated face; livid lips and cheeks; with intense heat and dryness of skin. After witnessing the last fearful paroxysm, a remission occurred, and with it the idea, that possibly *quinine might prevent another!* Certainly, it seemed if it were not for those terrible *exacerbations*, the local symptoms might be controlled. Was not the *remittent fever* which was destroying my patient, a mere *complication*, and of malarial origin? If so, quinine will control it,—*separate* it from the *local disease*, and we will have a *continued remission*. The idea was no sooner conceived, than my patient swallowed six grains of quinine; in one hour six more, which induced cinchonism. In one hour more, gave four grains quinine, and waited for the next paroxysm, due in half an hour. The half hour gone and a slight flush appears on the cheek, but it is *cool*. The *entire surface* is cool. No paroxysm returns, and the *remission continues*. Expectoration is established, the over burdened lung is relieved, my patient slowly recovers, and I have learned Dr. Upshur's *great truth*.

Since that time I have treated eight similar cases. They did not all assume the periodical character on the first, or even the second day; but the periodicity of *all* was clearly developed on the third, and of some, so early as the second day. In every case quinine was the sovereign remedy for the paroxysm, and all its formidable symptoms. The periodicity destroyed, there yet remained an uncomplicated pneumonia—a local inflammation of a manageable character, that readily yielded to expectorants and counter-irritants. On seeing how charmingly quinine acted in my first case, I was disposed to credit myself with having made an *innovation* on the treatment of pneumonia, but on referring to "Wood's Practice of Medicine," article, "Bilious Pneumonia," I found the disease and its treatment, both minutely described. I had read the article before, but had not discovered its importance. May not others have done the same? That article alone is worth the price of his book. Dr. Wood regards the cases above described, as pneumonia merely *modified* by malaria, and not of malarial origin. The limited experience I have had, fully warrants such a conclusion. On Oak Hill, six miles from this village, (Bradford, Steuben Co.,) where intermittents are never known, I treated three cases of pneumonia at the same time of the case reported above. They did not assume the paroxysmal character,—bore the lancet and nauseants well, and were not benefited, injured, or in any way sensibly affected by quinine,—although used as an experiment in two cases. In the valley, our pneumonia is complicated with a remittent fever of malarial origin—on the surrounding hills it is not. Quinine is *everything* in the treatment of pneumonia of the valley, and *nothing* in pneumonia of the mountain. Such are the facts relating to pneumonia in this locality, and its connection with malarial poisons.

From the above facts I infer,

1st. That pneumonia is not of malarial origin. It occurs frequently in regions unaffected by miasmatic poison. We know nothing of malaria, except from its effects upon the human organism—and they are specific—entirely characteristic of their cause. Pneumonia occurs without manifesting any of those characteristics, and wholly unaffected by quinine, the great specific in malarial disease. Again, pneumonia occurs, exhibiting in the highest degree the modifying influences of miasmatic poison, and that miasmatic influence is specifically controlled by quinine; but the malarial modifications being removed, we have *pneumonia yet remaining*—an uncomplicated local inflammation requiring its own appropriate treatment.

2d. Although quinine is not a *local antiphlogistic*, it is a *specific* in the worst cases of *malarial pneumonia*, where the *local inflammation* is not so extensive as to be incompatible with life; and the cases are few where the local disease is troublesome, after the attending miasmatic fever has been dismissed. The most fearful cases, lose their formidable character under the influence of quinine.

3d. That pneumonia may be influenced by malaria, and it may not. That in *malarial pneumonia*, quinine is of the greatest importance, and becomes

*absolutely necessary* to the cure of *bad cases.* That in non-malarial pneumonia, it is wasting a costly and most valuable remedy, to use quinine in its treatment.

